# Prevalence of and Factors Associated with Treatment Burden and Medication Adherence Among Primary Care Patients with Multimorbidity in Jeddah, Saudi Arabia: A Cross-Sectional Study

**DOI:** 10.7759/cureus.67514

**Published:** 2024-08-22

**Authors:** Yasser Y Khojah, Meaad A Bashawri, Nouf Y Khojah, Noha Saleh M Hassanien

**Affiliations:** 1 Preventive Medicine, King Abdullah Medical Complex, Jeddah, SAU; 2 Laboratory Medicine, Ministry of Health, Jeddah, SAU; 3 Physiotherapy, Hera General Hospital, Makkah, SAU; 4 Biostatistics, High Institute of Public Health, Alexandria University, Alexandria, EGY

**Keywords:** multimorbidity treatment burden, adherence to therapy, adherence to treatment, burden of treatment, multimorbidity

## Abstract

Background: Multimorbidity, the coexistence of multiple chronic conditions, presents significant challenges in treatment management and medication adherence. This study investigates the burden of multimorbidity and factors influencing treatment adherence among primary care patients in Jeddah, Saudi Arabia.

Methods: A cross-sectional study was conducted from November to December 2023, including 422 participants selected via stratified random sampling from 12 primary healthcare centers in Jeddah. Participants were adults aged 18 years or older with two or more confirmed long-term medical conditions. The Multimorbidity Treatment Burden Questionnaire (MTBQ) and General Medication Adherence Scale (GMAS) were used to measure treatment burden and medication adherence, respectively. Demographic variables were assessed for their influence on these outcomes.

Results: High treatment burden was reported by 55% of participants, while 21% reported medium burden, 15% experienced low burden and 9% no burden. Medication adherence was partial in 69% of participants, with 13% reporting high adherence, 13% good adherence, and 5% poor adherence. Statistically significant differences in MTBQ scores were observed based on marital status, education level, residence, occupation, and income. GMAS scores varied statistically significantly with marital status, education level, residence, occupation, income, and previous self-care education session attendance and MTBQ scores, with those having high burden reporting the lowest adherence scores.

Conclusions: Demographic factors, including marital status, education level, residence, occupation, and income, significantly influence multimorbidity treatment burden and medication adherence. A higher treatment burden was associated with lower adherence. Targeted interventions addressing these factors could improve treatment outcomes for patients with multiple chronic conditions.

## Introduction

Patients with chronic illnesses face significant challenges not only from their conditions but also from the extensive healthcare routines required to manage them. These routines typically involve taking medications, attending numerous medical appointments, monitoring health metrics, and adhering to specific dietary and exercise regimens [1,2].

The substantial burden of these healthcare activities can lead to a series of negative outcomes. Overwhelmed by their extensive regimens, patients often struggle to follow their prescribed treatments, leading to nonadherence [3-6]. This nonadherence can result in increased hospitalizations and higher mortality rates [7,8]. In response to poor health outcomes, physicians may intensify treatments, thereby increasing the burden on patients who are already struggling [1]. This cycle of increasing treatment demands can significantly impact patients' quality of life, as they must allocate more time, energy, and resources to maintaining their health [9].

Treatment burden refers to patients' perception of the effort needed to manage their health and the impact this effort has on their daily lives [10]. The current disease-centered approach to healthcare often demands that patients attend multiple appointments, implement lifestyle changes, self-monitor their conditions, and manage complex medication regimens. This creates a significant workload, leading to a high treatment burden. This burden is particularly heavy for patients with multimorbidity (multiple long-term conditions) and those who lack the capacity or support to integrate their treatment into their other life roles and responsibilities. The complexity of managing multiple conditions can lead to substantial treatment burden and impact medication adherence. Understanding the factors influencing these outcomes is crucial for developing effective interventions [11].

In Saudi Arabia, the prevalence of multimorbidity, based on self-reported data, was estimated to be 23.3% [12]. Limited research has explored the effects of multimorbidity on treatment adherence and the factors influencing these outcomes. This study aims to fill this gap by evaluating the treatment burden and medication adherence among primary care patients with multimorbidity in Jeddah, Saudi Arabia, and identifying the demographic variables that influence these factors.

## Materials and methods

Study design and setting

This cross-sectional study was conducted from November to December 2023 in Jeddah, Saudi Arabia. A multistage sampling technique was employed, dividing primary healthcare centers into four geographical areas: North, South, East, and West. Three centers were randomly selected from each region, resulting in 12 centers. A random sample of multimorbid patients attending each center during the study period was selected.

Participants

A total of 422 participants were included, representing a 73% response rate. Inclusion criteria were all adult patients aged 18 years or above with two or more clinically diagnosed long-term conditions (multimorbidity) and at least one clinical encounter in the past 12 months. Exclusion criteria included patients with a single morbidity, those living in a care home, those receiving palliative care, those with a mental health diagnosis (psychosis, schizophrenia, bipolar disorder), and patients with active cancer (recorded in the last three years).

Data collection

Data was collected using two questionnaires. The 10-item Multimorbidity Treatment Burden Questionnaire (MTBQ) assessed the physical, psychological, and social impacts of managing multiple chronic conditions [13]. The General Medication Adherence Scale (GMAS), consisting of 11 items, evaluated medication adherence indirectly by examining factors such as forgetfulness and difficulty understanding instructions [14]. Detailed items for both the MTBQ and GMAS are provided in Appendices A and B, respectively.

The reliability of the MTBQ (Cronbach’s alpha: 0.83) and the GMAS (Arabic version, Cronbach’s alpha: 0.87) indicated good internal consistency.

Additionally, sociodemographic data-including gender, age, occupational category, nationality, marital status, educational level, residence area, family income, and previous health education-were collected to identify factors influencing treatment burden and medication adherence.

The questionnaires were administered by trained researchers or healthcare workers. Participants also had the option to self-administer the questionnaires via an online form.

Statistical analysis

Descriptive statistics were used to summarize the demographic characteristics of the participants. Independent t-tests and ANOVA were conducted to assess differences in MTBQ and GMAS scores across demographic variables. Significant differences were reported with p-values less than 0.05. Due to violations of regression model assumptions, primarily related to low case numbers in some categories, regression analyses were not performed. All statistical analyses were performed using SPSS version 25.0 (IBM Corp, Armonk, NY, USA).

Ethical considerations

This study was conducted in accordance with ethical standards and was approved by the Ministry of Health Institutional Review Board (IRB) in Jeddah (IRB approval code A01772). Data collection was carried out anonymously to ensure the confidentiality and privacy of all participants. Informed consent was obtained from each participant prior to their inclusion in the study.

## Results

The study included 422 participants, with a slight majority being male (54%, n = 228). The median age was 39 years, with participants ranging from 18 to 88 years. Marital status varied, with 54.5% (n = 230) married, 30.6% (n = 129) single, 9.5% (n = 40) divorced, and 5.5% (n = 23) widowed. The majority of participants were Saudi nationals (82.5%, n = 248). Educational levels were notably high, with 70.6% (n = 298) holding a bachelor's degree or higher. Most participants resided in urban areas (87.4%, n = 369) and were employed full-time (55%, n = 232). Monthly income varied, with 40% (n = 169) earning more than SAR 12,000, while 6.4% (n= 27) earned less than SAR 1,000. Furthermore, 61.1% (n = 258) had not attended any self-care education sessions. Participants characteristics are presented in Table 1.

**Table 1:** Sociodemographic characteristics of participants

Characteristics	Frequency (%)
Total	422 (100%)
Gender	
Male	228 (54%)
Female	194 (46%)
Age in years, median (range)	39 (15-88)
Marital status	
Single	129 (30.6%)
Married	230 (54.5%)
Divorced	40 (9.5%)
Widowed	23 (5.5%)
Nationality	
Saudi	348 (82.5%)
Non-Saudi	74 (17.5%)
Education level	
Bachelor's degree or higher	298 (70.6%)
High school	90 (21.3%)
Intermediate	23 (5.5%)
Elementary	6 (1.4%)
Illiterate	5 (1.2%)
Residence	
Urban	369 (87.4%)
Suburban	45 (10.7%)
Rural	8 (1.9%)
Occupation	
Full-time employment	232 (55%)
Stay-at-home parent	80 (19%)
Retired	65 (15.4%)
Student	41 (9.7%)
Unemployed	4 (0.9%)
Monthly Income, Saudi Arabian Riyals (SAR)	
>SAR 12,000	169 (40%)
SAR 7,000-12,000	104 (24.6%)
SAR 4,000-7,000	81 (19.2%)
SAR 1,000-4,000	41 (9.7%)
	27 (6.4%)
Self-care education sessions attendance	
No	258 (61.1%)
Yes	164 (38.9%)

MTBQ Scores

**Figure 1:**
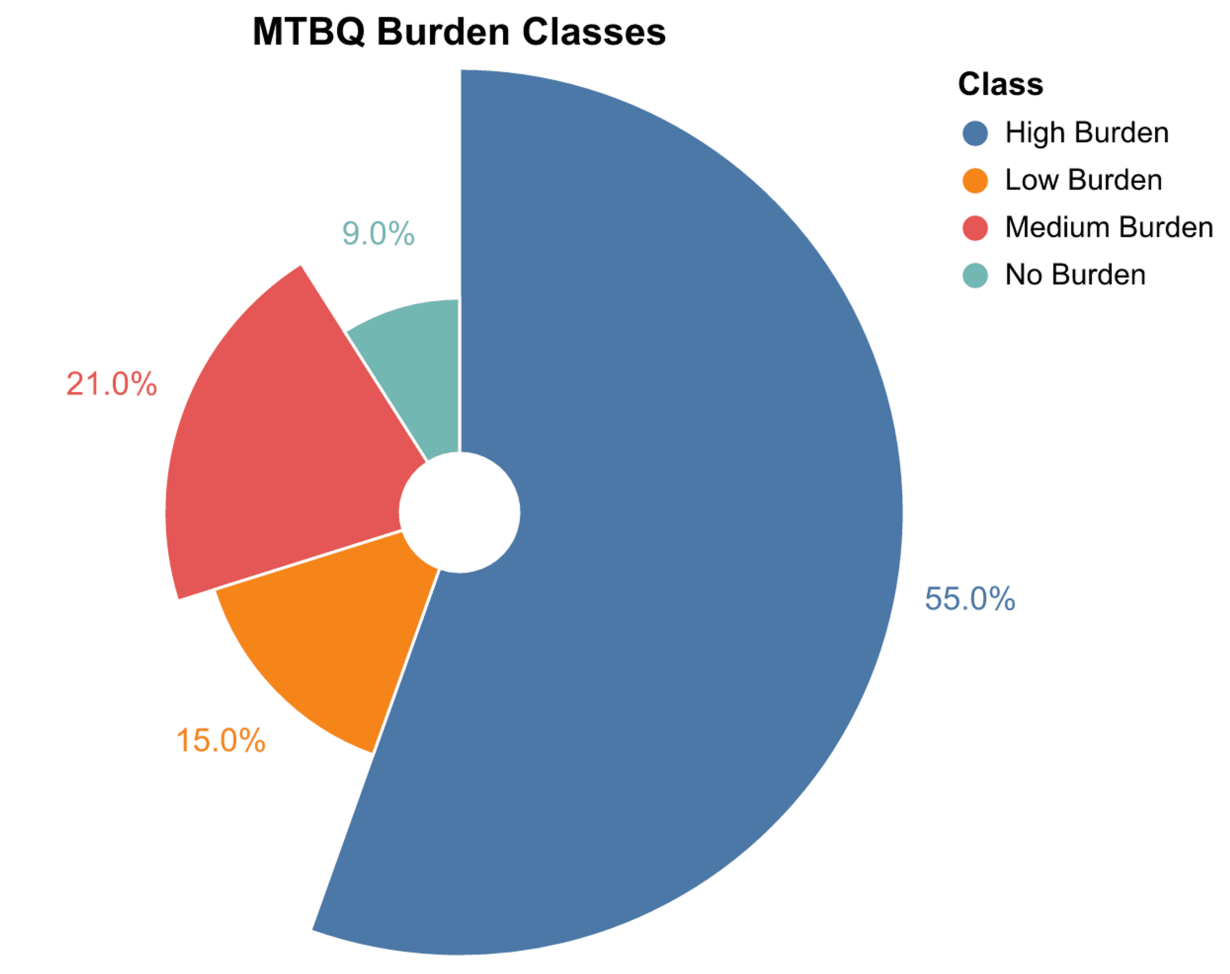
Distribution of MTBQ Burden Classes MTBQ: Multimorbidity Treatment Burden Questionnaire

**Figure 2:**
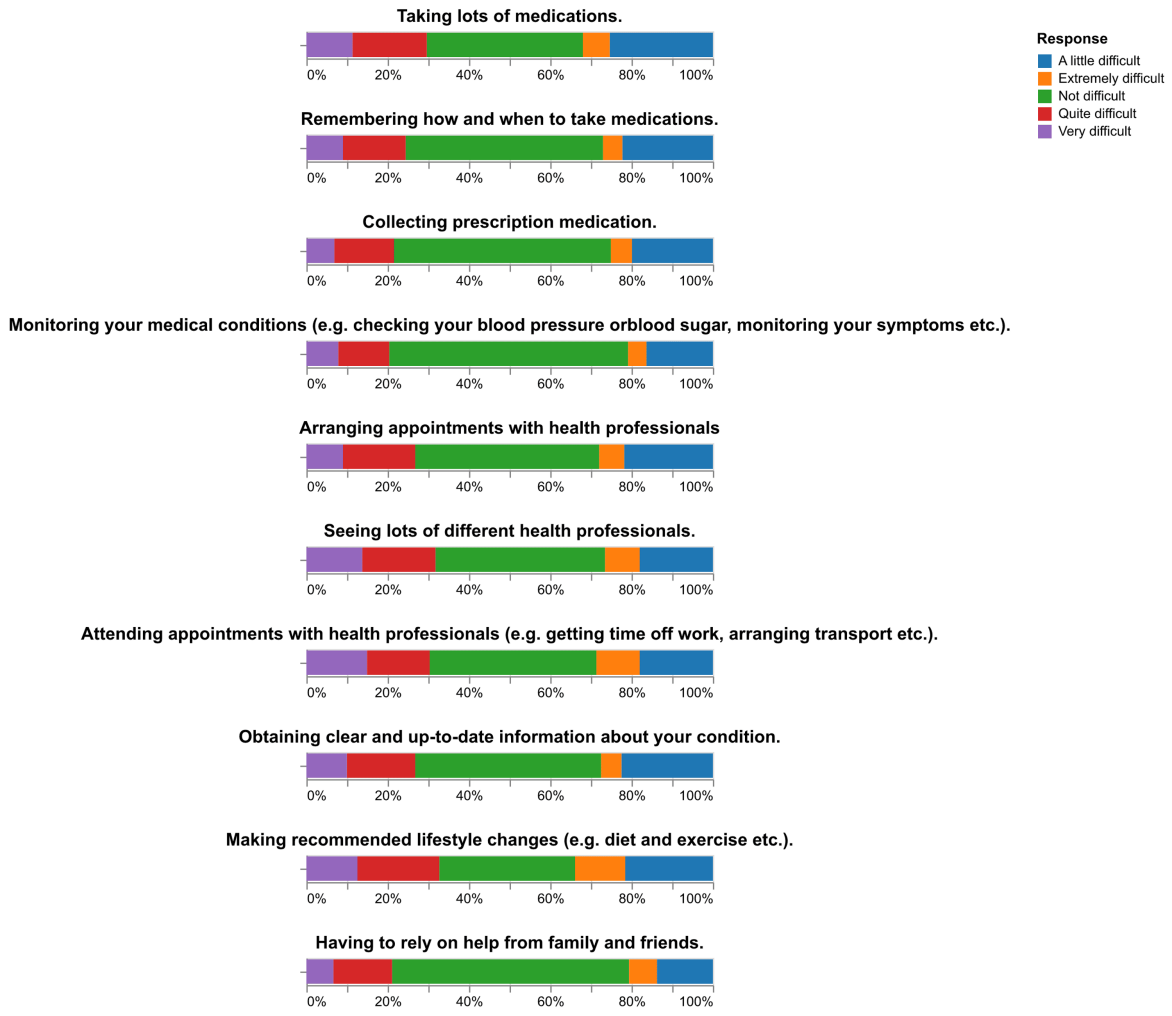
Distribution of Responses to Each Question in the MTBQ MTBQ: Multimorbidity Treatment Burden Questionnaire

The assessment of treatment burden using the MTBQ revealed varied levels of burden among the 422 respondents. The majority, 55% (n=232), reported experiencing a high burden. This was followed by 21% (n = 89) who reported a medium burden, 15% (n = 63) who reported a low burden, and 9% (n = 38) who reported no burden at all. These results are graphically presented in Figure 1. Additionally, the distribution of responses to each question in the MTBQ is illustrated in Figure 2, providing a detailed view of how different aspects of treatment burden were experienced by the participants.

The analysis revealed significant associations between the MTBQ scores and several demographic factors, as presented in Table 2. Marital status, education level, residence, occupation, and income significantly influenced treatment burden. Widowed participants had the highest burden (mean±SD: 44.13±20.29). Illiterate participants also reported higher burden scores (mean±SD: 57.00±31.69). Rural residents had the highest burden (mean±SD: 49.38±22.98), and unemployed individuals reported the highest burden among occupational categories (mean±SD: 70.00±16.96). Participants with lower income (< SAR 1,000) experienced higher burden (mean±SD: 36.02±21.97). No significant differences were found in MTBQ scores based on gender or nationality. Age correlated weakly positively with MTBQ scores ( Pearson’s *r* = 0.18).

**Table 2:** Subgroup analysis of MTBQ scores Post-hoc comparisons utilized the Bonferroni test following significant one-way ANOVA results MTBQ: Multimorbidity Treatment Burden Questionnaire

Characteristics	Mean ± SD	Significantly different from group	t/F	P-value
Gender	–	–	-0.867	0.387
Male	27.02 ± 21.33	–	–	–
Female	28.87 ± 22.25	–	–	–
Marital status	–	–	7.265	<0.001
A. Single	22.03 ± 22.43	B, D	–	–
B. Married	28.68 ± 20.87	A, D	–	–
C. Divorced	32.63 ± 19.29	–	–	–
D. Widowed	44.13 ± 20.29	A, B	–	–
Nationality	–	–	-1.583	0.117
Saudi	26.98 ± 20.76	–	–	–
Non-Saudi	32.03 ± 25.68	–	–	–
Education level	–	–	8.21	<0.001
A. Illiterate	57.00 ± 31.69	–	–	–
B. Elementary	50.00 ± 12.04	E	–	–
C. Intermediate	46.74 ± 22.57	E	–	–
D. High school	30.28 ± 19.40	–	–	–
E. Bachelor's and higher	24.75 ± 21.00	B, C	–	–
Residence	–	–	10.66	<0.001
A. Urban	25.62 ± 20.79	B, C	–	–
B. Suburb	42.50 ± 21.97	A	–	–
C. Rural	49.38 ± 22.98	A	–	–
Occupation	–	–	3.1	0.16
A. Employed	27.37 ± 21.70	B	–	–
B. Unemployed	70.00 ± 16.96	A, C, D	–	–
C. Student	15.18 ± 14.61	B, E	–	–
D. Retired	27.15 ± 19.04	B	–	–
E. Stay-at-home parent	34.28 ± 22.73	E	–	–
Income, Saudi Arabian Riyals (SAR)	–	–	2.817	0.025
A. < SAR 1,000	36.02 ± 21.97	–	–	–
B. SAR 1,000-4,000	34.02 ± 25.57	E	–	–
C. SAR 4,000-7,000	29.41 ± 23.25	E	–	–
D. SAR 7,000-12,000	27.81 ± 18.36	–	–	–
E. > SAR 12,000	24.36 ± 21.40	B, C	–	–

GMAS scores

**Figure 3:**
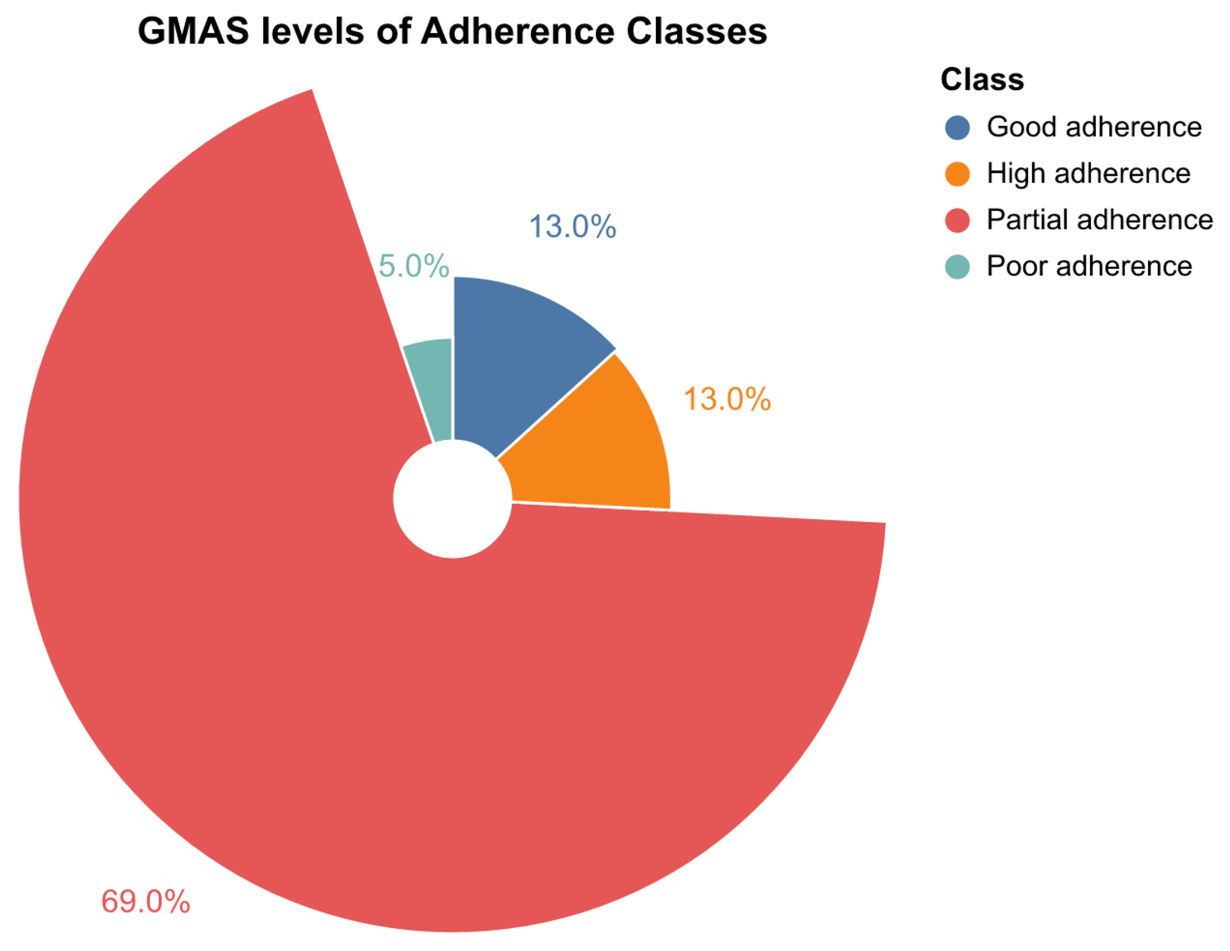
Distribution of GMAS levels of adherence classes GMAS: General Medication Adherence Scale

**Figure 4:**
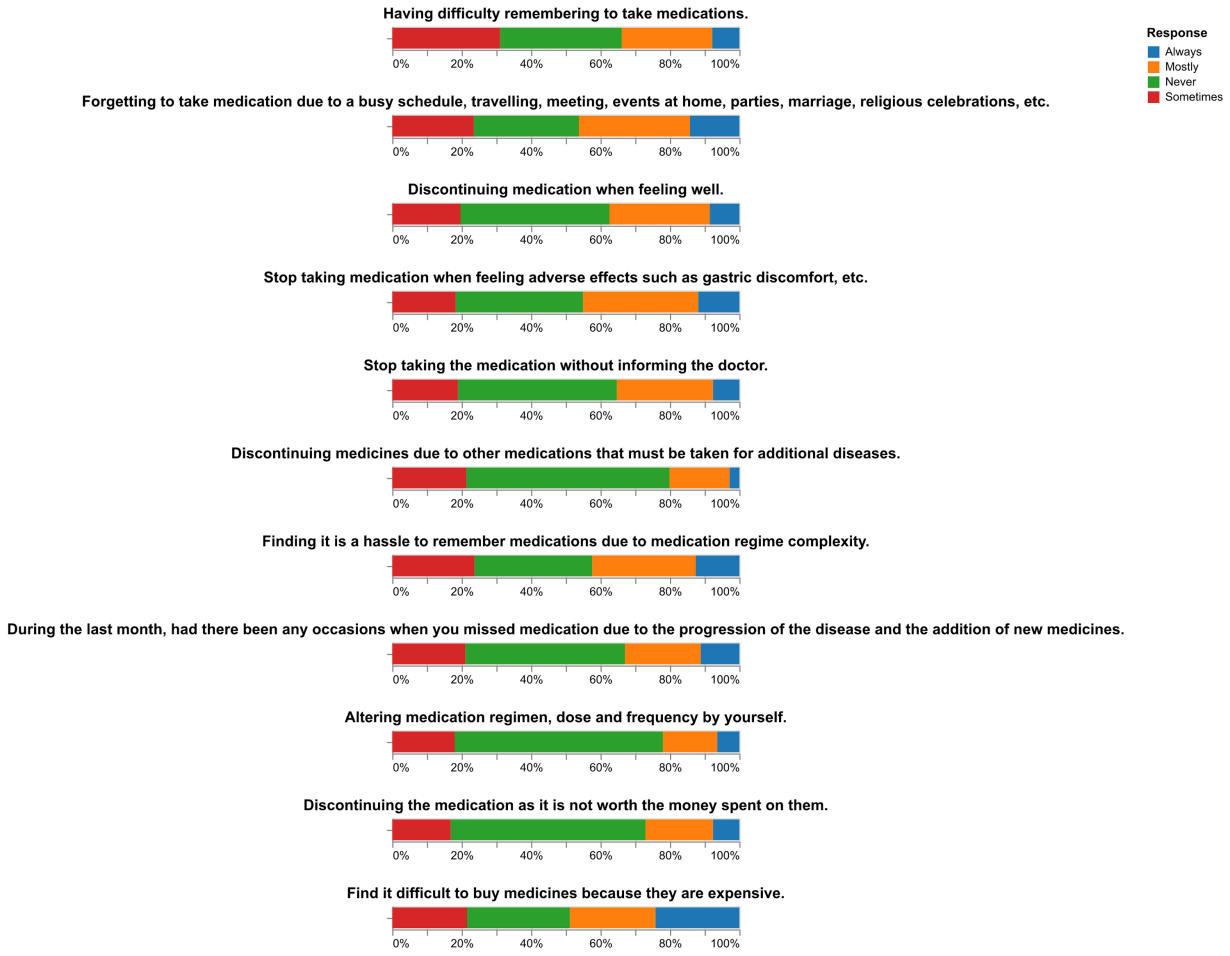
Distribution of responses to each question on the GMAS questionnaire GMAS: General Medication Adherence Scale

The GMAS scores indicated varying levels of medication adherence among the 422 participants. A majority of participants, 69% (n=291), were classified as having partial adherence. Good adherence was reported by 13% (n=55) of participants, while another 13% (n=55) demonstrated high adherence. Only 5% (n=21) of participants were classified as having poor adherence. These results are presented graphically in Figure 3. Additionally, Figure 4 presents the distribution of responses to each question on the GMAS, providing a detailed view of the participants' adherence behaviors.

The GMAS scores were significantly influenced by several demographic and socioeconomic factors, as presented in Table 3. Marital status showed notable differences, with single participants having the highest adherence scores (mean±SD: 23.36±6.739) compared to widowed participants who had the lowest scores (mean±SD: 17.04±7.339). Education level also played a significant role, with participants holding a bachelor's degree or higher demonstrating better adherence (mean±SD: 22.57±6.360) compared to illiterate participants (mean±SD: 15.20±8.843). Residence area influenced adherence, with urban residents showing higher scores (mean±SD: 22.24±6.474) than rural residents (mean±SD: 16.13±8.692). Occupational status was another significant factor, with students reporting the highest adherence (mean±SD: 23.66±5.425) and unemployed individuals the lowest (mean±SD: 14.50±3.000, P=0.016). Higher income levels were associated with better adherence, particularly for those earning more than SAR 12,000 (mean±SD: 22.83±7.031, P=0.025). Attendance at self-care education sessions significantly improved adherence scores (mean±SD: 23.04±6.109). Finally, participants classified with high treatment burden had the lowest adherence scores (mean ± SD: 18.96±6.239), indicating a negative correlation between treatment burden and medication adherence. Additionally, age correlated weakly negatively with adherence scores (Pearson's *r* = -0.16).

**Table 3:** Subgroup analysis of GMAS scores Post-hoc comparisons utilized the Bonferroni test following significant one-way ANOVA results GMAS: General Medication Adherence Scale; MTBQ: Multimorbidity Treatment Burden Questionnaire

Characteristics	Mean ± SD	Significantly different from group	t/F	P-value
Gender	–	–	0.842	0.4
Male	21.94 ± 6.686	–	–	
Female	21.39 ± 6.713	–	–	
Marital status	–	–	7.265	<0.001
A. Single	23.36 ± 6.739	B, C, D	–	–
B. Married	21.44 ± 6.460	A, D	–	–
C. Divorced	20.40 ± 5.956	A	–	–
D. Widowed	17.04 ± 7.339	A, B	–	–
Nationality	–	–	1.772	0.079
Saudi	21.98 ± 6.519	–	–	–
Non-Saudi	20.34 ± 7.368	–	–	–
Education level	–	–	8.21	<0.001
A. Illiterate	15.20 ± 8.843	E	–	–
B. Elementary	14.00 ± 7.616	D, E	–	–
C. Intermediate	16.96 ± 7.138	D, E	–	–
D. High school	20.84 ± 6.500	C, E	–	–
E. Bachelor's and higher	22.57 ± 6.360	A, B, C, D	–	–
Residence	–	–	10.66	<0.001
A. Urban	22.24 ± 6.474	B, C	–	–
B. Suburb	18.18 ± 6.756	A	–	–
C. Rural	16.13 ± 8.692	A	–	–
Occupation	–	–	3.1	0.016
A. Employed	21.91 ± 6.391	B	–	–
B. Unemployed	14.50 ± 3.000	A, C, D	–	–
C. Student	23.66 ± 5.425	B, E	–	–
D. Retired	21.86 ± 7.671	B	–	–
E. Stay-at-home parent	20.25 ± 7.047	C	–	–
Income, Saudi Arabian Riyals (SAR)	–	–	2.817	0.025
A. < SAR 1,000	20.96 ± 5.317	–	–	–
B. SAR 1,000-4,000	19.49 ± 7.750	E	–	–
C. SAR 4,000-7,000	20.83 ± 6.754	E	–	–
D. SAR 7,000-12,000	21.56 ± 5.648	–	–	–
E. > SAR 12,000	22.83 ± 7.031	B, C	–	–
Education sessions attendance	–	–	2.213	0.001
Yes	23.04 ± 6.109	–	–	–
No	20.83 ± 6.918	–	–	–
MTBQ classification	–	–	41.42	<0.001
A. No burden	26.68 ± 5.517	C, D	–	–
B. Low burden	26.32 ± 5.331	C, D	–	–
C. Medium burden	23.53 ± 5.488	A, C, D	–	–
D. High burden	18.96 ± 6.239	A, B, C	–	–

## Discussion

This study provides insights into the treatment burden and medication adherence among primary care patients with multimorbidity in Jeddah, Saudi Arabia. The findings revealed that a substantial proportion of patients experience high treatment burdens, and the majority demonstrate partial adherence to medication regimens. Specifically, 55% of participants reported high treatment burden, while 69% exhibited partial medication adherence. Importantly, the study found a significant interaction between treatment burden and medication adherence, with higher treatment burden associated with lower adherence. Several demographic factors, including marital status, education level, residence, occupation, and income, were significantly associated with both treatment burden and medication adherence.

The findings align with previous research highlighting the high treatment burden among patients with multimorbidity. For instance, a comprehensive review by Sav et al., involving more than 30 studies, found that patients with multiple chronic conditions often face significant treatment burdens, resulting in poor health and well-being, non-adherence to treatment, ineffective resource use, and burden on significant others [15]. Other research has also found significant associations between demographic factors and treatment burden and adherence to treatment, supporting the findings of this study [1,15-17].

The significant associations between demographic factors and treatment burden suggest that healthcare providers should consider a more personalized approach in managing patients with multimorbidity. Tailored interventions addressing specific needs based on marital status, educational background, and socioeconomic status could potentially enhance treatment adherence and reduce the overall burden. For example, educational programs targeted at patients with lower educational levels or those with limited health literacy may improve their understanding of treatment regimens and encourage better adherence.

The findings also indicate the importance of self-care education sessions, as patients who attended these sessions demonstrated better medication adherence. This suggests that integrating comprehensive self-care education into routine patient care could be beneficial, particularly for those managing multiple chronic conditions.

Future studies could explore longitudinal designs to establish causal relationships between treatment burden, medication adherence, and demographic factors. Additionally, qualitative research could provide deeper insights into the specific challenges faced by patients with multimorbidity, informing the development of targeted interventions. Investigating the role of cultural and social factors in different regions of Saudi Arabia and other Middle Eastern countries could also provide a more comprehensive understanding of how these factors influence treatment burden and adherence.

The cross-sectional design precludes causal inferences, limiting the ability to determine the directionality of the relationships observed. Additionally, the reliance on self-reported data may introduce response bias, as patients might underreport or overreport their adherence behaviors and perceived burdens.

A significant limitation is the small number of participants in certain demographic categories, such as those in rural areas or the unemployed. This might have affected the results by reducing the statistical power and the ability to detect significant differences in these subgroups. The small sample sizes in these categories could lead to less reliable estimates and wider confidence intervals, potentially obscuring true associations or exaggerating the influence of outliers.

## Conclusions

In conclusion, this study underscores the significance factors that are influencing medication adherence and the burden of disease for instance, marital status, education level, residence, occupation, and income. A notable finding is the interaction between adherence and treatment burden, where a higher burden is associated with lower adherence. By addressing these factors and the interplay between adherence and treatment burden, tailored interventions can improve patient health outcomes and quality of life. Healthcare providers and policymakers should focus on personalized care approaches and integrate self-care education to better support patients managing multiple chronic conditions.

## Appendices

Appendix A 

**Table 4:** The MTBQ MTBQ: Multimorbidity Treatment Burden Questionnaire

We are interested in finding out about the effort you have to make to look after your health and how this impacts on your day-to-day life. Please tell us how much difficulty you have with the following: (Please tick the box that most applies to you)						
	Not difficult	A little difficult	Quite difficult	Very difficult	Extremely difficult	Does not apply
1. Taking lots of medications						
2. Remembering how and when to take medication						
3. Paying for prescriptions, over the counter medication or equipment						
4. Collecting prescription medication						
5. Monitoring your medical conditions (e.g. checking your blood pressure or blood sugar, monitoring your symptoms, etc.)						
6. Arranging appointments with health professionals						
7. Seeing lots of different health professionals						
8. Attending appointments with health professionals (e.g. getting time off work, arranging transport etc.)						
9. Getting health care in the evenings and at weekends						
10. Getting help from community services (e.g. physiotherapy, district nurses etc.)						

Appendix B 

**Table 5:** The GMAS GMAS: General Medication Adherence Scale

Patient behavior factors	
1-	Having difficulty remembering to take medications.
2-	Forgetting to take medication due to a busy schedule, travelling, meeting, events at home, parties, marriage, religious celebrations, etc.
3-	Discontinuing medication when feeling well.
4-	Stop taking medication when feeling adverse effects such as gastric discomfort, etc.
5-	Stop taking the medication without informing the doctor.
Comorbidity and pill burden factors	
6-	Discontinuing medicines due to other medications that must be taken for additional diseases.
7-	Finding it is a hassle to remember medications due to medication regime complexity.
8-	During the last month, had there been any occasions when you missed medication due to the progression of the disease and the addition of new medicines.
9-	Altering medication regimen, dose and frequency by yourself.
Cost-related factors	
10-	Discontinuing the medication as it is not worth the money spent on them.
11-	Find it difficult to buy medicines because they are expensive.
